# Therapeutical Notes

**Published:** 1900-06-09

**Authors:** 


### THERAPEUTICAL NOTES.

### Epistaxis.

Rendu recommends insifflations of a powder of this type:—

Antipyrin, part 0·5.
Tannin, part 1.
Powdered sugar, parts 10.

Small quantities of the above blown up the nostril have a remarkable styptic effect.—*Med. Record*, Jan. 13th.

### Broncho-pneumonia in Children.

℞. Ammonii acetatis, gr. viii.

Sodii benzoatis, gr. xxx.

Oxymel scillæ, ʒijss.

Syrupi pruni virgin, ℥i.

Aquæ distillatæ, ℥iijss.

M. S. A teaspoonful or more every two hours according to the age of the child.—Perier, *Med. Record*, Jan. 13th.

### Restlessness of the Aged.

Dr. C. Dukes recommends nitroglycerine, gr. 1-100, or erythrol tetranitrate, gr. ½-1, for the relief of this condition. The effects of the latter last longer, and therefore it need not be repeated so often. He finds that the insomnia by night, and the constant moving about by day are quickly cured by these vaso-dilators. Dr. Bradbury, in his recent lecture, remarks that nux vomica or strychnine is most helpful in treating the insomnia of old age.—*Brit. Med. Jour.*, December 2nd.

### Sciatica.

Klemperer gives the results of treatment with methylene blue in 27 cases. In eight the remedy failed, in six the cure was rapid and complete, while in the rest the relief was marked, but the progress was slow. In a few cases the remedy seemed to cause some stomach disturbance, and in others led to pain in micturition, which could, however, be prevented by the addition of an equal quantity of nutmeg to the dose of methylene blue. There are, he thinks, many obstinate cases where the remedy may be of use, which resist all ordinary remedies.—*Merck's Archives*, January.

